# Use of GPS for Older Adults to Decrease Driving Risk: Perceptions from Users and Non-Users

**DOI:** 10.3390/geriatrics5030060

**Published:** 2020-09-22

**Authors:** Anne E. Dickerson

**Affiliations:** Department of Occupational Therapy, East Carolina University, Greenville, NC 27858, USA; dickersona@ecu.edu

**Keywords:** older adults, driving, GPS technology, community mobility

## Abstract

Community mobility is important for social participation and quality of life. Thus, it is important to sustain older adults in their communities by supporting their ability to drive as long as possible. Use of global positioning system (GPS) technology may provide such support. This descriptive study examined 89 healthy community older adults’ perspective on using and programming a GPS after using it for wayfinding to unfamiliar destinations. Participants were equally divided between two age groups (60s, 70s) and familiarity with GPS (familiar, unfamiliar). The results showed age differences in problems following GPS directions and those who were familiar found it significantly easier to use. The majority of the unfamiliar group indicated an increased interest in using GPS and were significantly more interested in training to use a GPS. Preference for learning how to use a GPS included in-person delivery and practice with troubleshooting, using the menus and changing routes as topics critical for training. The implications of these results are discussed.

## 1. Introduction

Driving is and will continue to be the preferred method of community mobility for older adults. This is especially true for the majority of older adults who live in suburban and rural communities [1]. In addition, community mobility is critical for maintaining physical and mental health and quality of life [2] and essential as a means to engage in activities outside the home in order to enhance and support social participation [3]. Thus, to support our older adult’s wellbeing, professionals in both healthcare and transportation must work together to provide transportation mobility throughout the life span [4], especially for those most vulnerable, when driving is no longer possible and public methods of transportation are limited or not available. Transportation equity [4] will become more of a public health issue as the US population ages with those over the age of 80 becoming the fastest growing age group. This issue has been highlighted by the COVID-19 pandemic. Older adults without the ability to drive and who have limited social contacts are at far greater risk of not getting their basic needs met without public awareness and assistance. In fact, the social isolation of older adults in the context of COVID-19 has been identified as one of the most affected health outcomes [5].

Regardless of the current crisis, there is a long term need to maintain community mobility and social participation of older adults who are demonstrating some normal aging changes, including slower cognitive processing. Simplistically, there are two options: provide alternation transportation options (e.g., walking, cycling, bus, taxi, transportation network options, para-transit) or support older adults’ preferred option of mobility—driving. Unfortunately, the structure of the North America’s car dependent environment provides less opportunities for active transportation modes [6] and the expansion of transportation alternatives beyond most city limits is cost prohibitive. Moreover, although advanced driver assistance systems, including collision avoidance, have been shown to decrease crashes, it will be at least twenty years before widespread availability will actualize this potential for the wider population [7,8].

Thus, while there is a clear need for healthcare practitioners to require driving cessation for medically at-risk adults (of any age) who are genuinely unfit to drive, these same healthcare providers must consider strategies to decrease risk but maintain community mobility for those aging drivers with limited alternative options. In fact, O’Neill et al. [4] argue there may be an overemphasis on older driver safety. With the safety of vehicles increasing and crash rates for older drivers decreasing [9], practitioners must balance the risk of a crash, which is relatively rare [10], with the over-restriction of older adults that may cause a decrease in life-space mobility and quality of life. One potential strategy for the challenging aspect of driving—wayfinding—is through navigational technology to assist when searching a destination in an unfamiliar environment. 

Considering Michon’s [11] driving behavioral hierarchy, older adults retain their operational and tactical levels of driving behavior, that is, they can steer, use the pedals, and follow the rules of the road often without a lot of conscious awareness. It is only when challenged at the strategic level, for example, needing to navigate a new route, that older drivers will demonstrate impairment in performance compared to younger drivers due to the increased cognitive demands associated with navigating unfamiliar environments [12]. In fact, research has shown that older drivers do show slower processing speeds and poorer attentional abilities in driving performance [13] and have more difficulty wayfinding during route finding tasks than younger adults [14,15]. However, slower processing does not mean older adults are unfit to drive. Moreover, if older drivers used navigational systems to support their wayfinding tasks, it could potentially decrease the driving risk during strategic tasks. 

As early as 1989, researchers have examined the determinants of technology acceptance resulting in the development of the Technology Acceptance Model (TAM) [16,17]. The model postulates that two beliefs, perceived usefulness and perceived ease of use, underlie the acceptance of technology. With a focus more on driver safety issues, Eby and Kostynick found drivers using GPS outperformed those using paper directions [18]. In a recent study, driving performance with GPS was also better than paper directions on unfamiliar routes [19]. However, there have been multiple studies that suggest drivers may be less safe when using navigational devices [20,21,22,23]. It may be that older adults take longer to program and process the information of the GPS while driving [24]. More recent work examined age difference with navigation performance in a virtual maze [25]. In their results, older adults had significantly more navigation errors, suggesting inefficiency in route learning. However, significantly more errors in route learning still does not equate to being unfit to drive. Additionally, a study examining GPS use found that even with a short training session, there was substantial improvement in destination entry performance [26].

Most importantly, a recent study demonstrated that driving performance was aided by the use of an electronic navigational system or GPS [27]. Specifically, 80 older adults between 60 and 79 years old each drove two unfamiliar routes, one with paper directions and the other using a pre-programmed GPS. Half of each age group was familiar with GPS and the other half was unfamiliar. Across all participants, participants exhibited better driving performance using the GPS over the paper directions. Not surprisingly, there was also an age difference with those in the 60s performing better than those in their 70s with no interaction effects. This study’s results suggest that navigational support has the potential to benefit older adults by improving driving performance and therefore safety [27].

However, in a survey of older adults in Australia [28], many older adults were not sure how GPS worked or thought they may be too complicated to operate. When concerning technology, barriers include user interface and user experience preferences of older adults [29]. Thus, the TAM may be a useful modeling for understanding the technology acceptance behavior of older adults, paired with the characteristics, abilities and problems experienced by older adults [30]. Specifically, this study was designed to examine older adults’ perspective on the usefulness of a GPS for wayfinding as well as the ease of programming (i.e., ease of user interface) a GPS after experiencing both in a structured study. Specifically, we explored whether non-users of GPS differed from users of GPS and older adults in their 60th decade experienced GPS differently from those in their 70th decade when asked about experiences using and programming a GPS. In addition, in preparation for the second experiment in Thomas et al. [27], questions were included about interest and strategies for training.

## 2. Methods

### 2.1. Design

This was a descriptive survey using Qualtrics^®^ on an iPad that was administered immediately after each participant completed only Experiment 1 in Thomas et al. [27]. All participants signed a separate consent form for the experimental study [27] and one for this study (UMCIRB 15-000017), both were approved by East Carolina University’s University and Medical Center Institutional Review Board.

### 2.2. Participants

Eighty-nine healthy older adults living in the community, who drove regularly, completed the survey. The participants were recruited to participate in a study designed to determine if the use of GPS assisted with driving performance (see Thomas, et al., 2020 [27], Experiment 1). This survey also included the nine pilot study participants for a total of 89. Of the 89, 45 were between 60–69 years of age (75% female) and 44 were between 70–79 years of age (62.5% female). When asked, 45 participants were classified as “familiar” with using GPS regularly and/or comfortable using it occasionally. The remaining 44 participants were considered “unfamiliar” because they indicated they: (1) may have used, but were not confident using GPS, (2) may have tried GPS, but not comfortable using it, or (3) never used GPS.

### 2.3. Procedure

The survey was designed and piloted with content experts resulting in 13–16 questions, depending on responses to yes/no questions (i.e., a “yes” triggered an additional question). The questions asked respondents: (1) if they had difficulties using the GPS on the road, (2), if yes, what were those difficulties? (3) preferences towards the GPS or paper directions, (4) if they had difficulties programming the GPS, (5) if yes, what were those difficulties? (6) their interest in purchasing a GPS, and (7) desire and types of training preferred to learn how to use a GPS.

As descripted in Experiment one [27], each participant drove four routes: (1) one familiar route, (2) one unfamiliar routes using paper directions and/or a map, (3) one unfamiliar route using a pre-programmed GPS, and (4) a second route using GPS. Routes 2 and 3 were similarly designed; unfamiliar routes were counterbalanced with each other as well with the order of GPS and paper directions. With each route taking approximately 20 min, each participant experienced following the GPS directions for approximately 40 min. Upon return to the laboratory, all participants were required to attempt programming the GPS for nine destinations (see details in Thomas et al., 2020 [27]).

Immediately after completing the programming, each participant was asked to answer the survey questions using an iPad by a research assistant, who entered the participants’ identification code and remained available to ensure there were no problems completing the survey. Data were downloaded from Qualtrics^®^ into a spreadsheet. Data analysis included descriptive statistics and Chi square analysis to compare perceptions of using and programming GPS between the contrasting groups (e.g., 60 years old versus 70 years old, unfamiliar with GPS versus familiar with GPS). Significance level was set at 0.05 and SPSS, Version 26 was used to analyze the data.

## 3. Results

The research questions examined each participant’s perception of the: (1) usefulness of the GPS for wayfinding, (2) ease of the user interface to program the GPS, and (3) interest and strategies for training. The results were compared between non-users of GPS from users and if older adults in their 60th decade experienced GPS differently from those in their 70th decade.

### 3.1. Usefulness

Of the 89 participants 19 indicated they had problems following the GPS directions on the drives. Given a list of potential problems, no single problem was consistently identified. Only two participants indicated that it was distracting and two indicated the GPS was “wrong”. Table 1 shows the significance between the age groups (χ^2^ (1, *n* = 89) = 5.6, *p* = 0.02). There was no significant difference in terms of familiarity with the GPS.

Ninety-one percent of the participants agreed or strongly agreed when asked to state the degree of confidence that the directions given by the GPS were trustworthy. Specifically, 53.9% (*n* = 48) strongly agreed; 37.1% (*n* = 33) agreed; 6.7% (*n* = 6) were neither agreed or disagreed; and 2.2% (*n* = 2) strongly disagreed. There were no significant differences for age or familiarity. When asked if they found the GPS distracting, almost all responded negatively: 62.9% (*n* = 56) indicated not at all; 29% (*n* = 26) indicated only slightly; 1.1% (*n* = 1) indicated moderately; and 1.15% (*n* = 1) indicated very.

Table 2 illustrates the comparison between the familiar and unfamiliar groups when asked which method (GPS or printed/map) was easier to use to navigate the routes. There was no significant difference in age, but a significant difference in familiarity (χ^2^ (1, *n* = 89) = 9.26, *p* = 0.01). Notably, 61% of the participants who were unfamiliar felt the GPS was easier, 18% felt it was about the same as printed, and only 20% indicated that printed directions were easier to follow.

Table 3 shows the significant difference between the groups in terms of familiarity with GPS in responding to which method they preferred based on this recent experience, the GPS or printed directions (χ^2^ (1, *n* = 89) = 9.49, *p* = 0.009). There was no difference based on the age groups. Unexpectedly, over half (*n* = 23, 52%) of the unfamiliar participants preferred the GPS over the printed map.

### 3.2. Ease of User Interface

Problems entering the addresses during the programming task were reported by 38 (42.7%) participants. Most common problems included: the keyboard was confusing (*n* = 15), touching the correct place on the screen (*n* = 14), the menus were confusing (*n* = 14), I never got to the destination (*n* = 13), it took too long to enter the destination (*n* = 12), I got the sequence of city, street and number wrong (*n* = 10). There was a significant difference between the familiarity groups (χ^2^ (1, *n* = 89) = 12.4, *p* = 0.001) with the unfamiliar participants having the most problems. There was no significant difference between the age groups.

Based on this recent experience (i.e., using the GPS on the routes), 31 or 70.5% of the unfamiliar group indicated an increased interest in using GPS. Table 4 illustrates the comparison between the two groups. There was no difference between age groups.

### 3.3. Training

There was a significant difference in interest in receiving training between the two familiarity groups on the use of a GPS (χ^2^ (1, *n* = 89) = 16.07, *p* = 0.003), but not between the two age groups. The majority (*n* =33, 73.3%) of the unfamiliar participants were either moderately or very interested in receiving training on how to use the GPS. For the rest of the questions on training, the participants who were not interested at all in training (*n* =23, familiar = 19, unfamiliar = 4) were not shown the questions.

**Figure 1:**
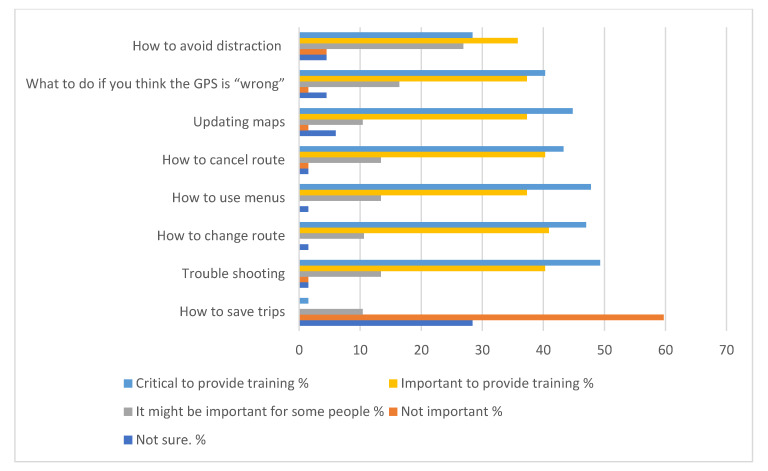
Topics for training concerning user interface while navigating and their level of importance. *n* = 66.

**Figure 2:**
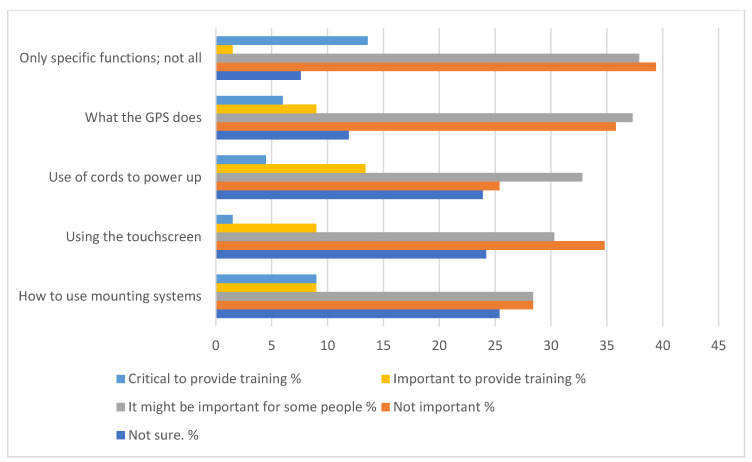
Topics for training concerning user interface with programming and their level of importance. *n* = 66.

When asked the best delivery method for this training (only one choice was allowed), the responses were: in person by an instructor (*n* = 22, 33.3%); on the GPS itself (*n* = 20, 30.3%); watch a video (*n* = 12, 18.2%); by a knowledgeable friend (*n*= 6, 9.0%); use computer as a reference (*n* = 3, 4.5%); a manual (*n* = 2, 3%); and other (*n* = 1, 1.5%). One “other” answer was a combination of instructor, hands on, watching a video, and a manual with better instructions. When asked how much time participants would be willing to devote to training, the responses were: 30–60 min (*n* = 23, 34.8%); 30 min or less (*n* = 17, 25.8%); however long it takes (*n* = 18, 27.3%); up to 2 h (*n* = 7, 10.6%); and 1 (1.5%) indicated no time. Finally, Figure 1 and Figure 2 shows the responses when asked what topics the GPS training should focus on as well as the degree of importance.

## 4. Discussion

The aim of this study was to examine older adults’ perception of the usefulness and ease of user interface after an experience of using GPS for wayfinding on unfamiliar routes and finding nine diverse destinations on a stationary device. A survey of training needs was also conducted. Overall, the experience was positive for use of the GPS for wayfinding, although programming the GPS was difficult for close to half of the participants, particularly for those who were unfamiliar.

In terms of perception of usefulness, over 91% of the participants believed the directions given by the GPS were trustworthy and not distracting. This confirms earlier research that navigational technology is perceived by older adults as helpful for wayfinding, with minimal distractions [31]. These results, however, are contrary to earlier studies reporting concerns as to the safety and utility of in-vehicle navigational technology in relation to older drivers [32,33]. However, it is important to consider technological advances. The design of GPS devices has improved with significant design enhancements (e.g., specific names of streets instead of vague turns, larger screens) since these previous studies. Therefore, discrepancies from these previous studies are understandable.

Only 19 or 21.3% reported problems with the GPS on the drives. Moreover, those reporting problems were not primarily in the unfamiliar group, indicating that following the GPS is may be relatively easy to use regardless if one is familiar or not. This is supported by the fact that the Thomas et al. [27] study showed fewer driving performance mistakes when using the GPS over the printed directions. Nevertheless, results also indicated that the older adults in their 70s had more difficulties than those in their 60s, supporting earlier age-related deficits in navigational strategies [34]. However, one unfamiliar participant in the 70s age group explained after the drive: “*I would sometimes turn too early because the direction to turn comes before the actual turn; I learned to look at the picture better*”. This suggests that while agreeing that they had a problem, it was clear learning can and did occur.

However, not all drivers in the unfamiliar group selected GPS as easier than printed directions for the wayfinding task. While not surprising, there were more unfamiliar drivers who found paper directions easier, it was noteworthy that 27 or 61% of the 44 unfamiliar drivers indicated the GPS was easier. Similarly, 23 or 52% of the unfamiliar drivers preferred the GPS after using both types of methods on similar routes. This outcome suggests that experience of the usefulness of technology may be necessary to understand and appreciate its value, supporting previous research [35,36,37,38,39]. It is important to note that the GPS routes were preset and, if followed correctly, there were no issues in arriving at the destinations. Even if an error occurred (i.e., typically a missed turn), the GPS recalculated the driver back. Conversely, the paper directions required much more cognitive effort to find streets and get back on track if an error occurred. However, in two cases, the drivers incorrectly followed the GPS directions, resulting in one intervention to get back on route and the other making a risky maneuver.

Participants in the unfamiliar group disproportionately had difficulty with programming the GPS, citing various issues rather than any one issue. Thus, it is not surprising that the majority in the unfamiliar group were interested in learning to use a GPS. However, it was not expected that this relatively short experience of using the GPS would significantly change the mindset of 70% of the older adults in the unfamiliar group who responded that they had a greater interest in using a GPS. Anecdotally, one of the most frequent questions after completion of the study was how much a GPS cost and where one could be purchased. This result should motivate both practitioners, family members, and lifelong educators to build and promote active in-vehicle learning for improving driving performance and, accordingly, driver safety.

### 4.1. Training

This study provides information on how best to offer training for learning how to use a navigational device. Clearly, the older adults in this study valued learning by face-to-face instruction with hands on learning. This is similar to earlier findings on learning preferences on how to use technology [40]. However, in this prior study, the top choice for learning was text (e.g., manuals) at 33% with one-to-one and trial and error (i.e., hands on) being significantly lower choices at 18% and 17%, respectively [40]. Of these 66 participants, only two participants (3%) selected a manual. It may be that older adults’ preferences have changed since the 2008 study as a matter of environmental availability; “how to” manuals are frequently online rather than printed with most products. This discrepancy might be an area of further research.

Most of the participants who indicated they wanted training were willing to devote a reasonable amount of time to learning how to use it. In terms of the topics for learning, participants indicated troubleshooting as most important, with needing to change routes, menus, and updating as critical tasks to learn. In fact, these data were used to develop six short videos for training using a GPS. Thomas et al. [27] included a second experiment with 60 older adults, all unfamiliar with GPS, assigned to three training groups: GPS video training, GPS video training and one-to-one instruction, and a placebo video training. Training was a significant predictor of the differences, with the three groups showing differences in terms of data entry errors [27]. As expected, participants in the one-to-one training had the best performance. These participants had the fewest numbers of errors and faster data entry time, suggesting that difficulties experienced when trying to use technologies may be diminished with training.

### 4.2. Limitations

The main limitation of this study was the screening tool for familiarity of GPS not being discriminate enough. A more specific tool examining the participant’s computer technology skills should have been included, as a few individuals who were “unfamiliar” actually easily used their computer knowledge to program the GPS competently. However, overall, this did not appear to be true for the majority of the unfamiliar participants. There were also participants, especially in the unfamiliar group, who became frustrated with the programming tasks and may not have given the survey sufficient attention, as in the last task. On the other hand, the value of immediate perceptions overshadows this limitation and the results are primarily positive in relation to older adults’ experience with GPS.

### 4.3. Implications

While this is only a survey on older adults’ perceptions, this study supports previous research that active and in-vehicle experience is essential for learning and promoting driving safety [41]. In this study, older adults who were not experienced with technology learned to appreciate how navigational technology may assist them in decreasing their driving risk and potentially extending their driving lifespan. This was true for even those older adults without previous experience and/or low experience with technology who had difficulty with data entry issues. Thus, education and training are essential for older adults to successfully use navigational devices [42,43] as well as other technology [29,40] and this education will be most successful with hands on learning and practice with an actual device [27].

Certainly, there are older adults with significant medical conditions, in particular dementia, who will be unable to learn how to use and benefit from technology. However, as O’Neill et al. [4] argues, over-restriction of older adults may cause a decrease in life-space mobility and therefore quality of life. With the safety of vehicles increasing and crash rates for older drivers decreasing [9], healthcare providers need to assist their clients with sensible and equitable decisions about driving and consider options, such as GPS technology, when appropriate, to decrease risk and ensure individuals have equitable access to transportation options to ensure quality of life.

## Figures and Tables

**Table 1:** Comparison of age groups for problems with GPS on drives (*n* = 89).

	Yes *n* (%)	No *n* (%)	χ^2^	*p*
Age: 60s	5 (10)	40 (35)	5.61	0.02
Age: 70s	14 (9)	30 (35)		

**Table 2:** Comparison of familiarity groups for ease of use—GPS or printed directions. (*n* = 89).

	Familiar *n* (%)	Unfamiliar *n* (%)	χ^2^	*p*
GPS was easier	39 (43.8)	27 (30.3)	9.26	0.01
About the same	5 (5.6)	8 (9.0)		
Printed easier	1 (1.1)	9 (10.1)		

**Table 3:** Comparison of familiarity groups based on preference for use—GPS or printed directions. (*n* = 89).

	Familiar *n* (%)	Unfamiliar *n* (%)	χ^2^	*p*
GPS	37 (41.6)	23 (25.8)	9.49	0.009
No preference	5 (5.6)	10 (11.2)		
Printed	3 (3.4)	15 (16.9)		

**Table 4:** Comparison of familiarity groups when asked about interest in using a GPS. (*n* = 89).

	Familiar *n* (%)	Unfamiliar *n* (%)	χ^2^	*p*
Interest is Lower	2 (2.2)	1 (1.1)	16.07	0.003
Interest is the same	30 (33.7)	12 (13.5)		
Interest is higher	13 (14.6)	31 (34.8)		

Note: A five-point scale was collapsed (1 = much lower, 2 = slightly lower, 3 = about the same, 4 = higher, 5 = much higher).
